# Self-Assembling Drug Formulations with Tunable Permeability and Biodegradability

**DOI:** 10.3390/molecules26226786

**Published:** 2021-11-10

**Authors:** Gulnara Gaynanova, Leysan Vasileva, Ruslan Kashapov, Darya Kuznetsova, Rushana Kushnazarova, Anna Tyryshkina, Elmira Vasilieva, Konstantin Petrov, Lucia Zakharova, Oleg Sinyashin

**Affiliations:** Arbuzov Institute of Organic and Physical Chemistry, FRC Kazan Scientific Center, Russian Academy of Sciences, Arbuzov Street 8, 420088 Kazan, Russia; leysan.vasileva@iopc.ru (L.V.); rusl701@yandex.ru (R.K.); darya.kuznetsova@iopc.ru (D.K.); ruwana1994@mail.ru (R.K.); ane4ka1567@mail.ru (A.T.); elmiravasilyeva@iopc.ru (E.V.); kpetrov2005@mail.ru (K.P.); luciaz@mail.ru (L.Z.); oleg@iopc.ru (O.S.)

**Keywords:** biological barrier, surfactant, nanocontainer, nanoparticle, micelle, niosome, transfersome, chitosome, invasome

## Abstract

This review focuses on key topics in the field of drug delivery related to the design of nanocarriers answering the biomedicine criteria, including biocompatibility, biodegradability, low toxicity, and the ability to overcome biological barriers. For these reasons, much attention is paid to the amphiphile-based carriers composed of natural building blocks, lipids, and their structural analogues and synthetic surfactants that are capable of self-assembly with the formation of a variety of supramolecular aggregates. The latter are dynamic structures that can be used as nanocontainers for hydrophobic drugs to increase their solubility and bioavailability. In this section, biodegradable cationic surfactants bearing cleavable fragments are discussed, with ester- and carbamate-containing analogs, as well as amino acid derivatives received special attention. Drug delivery through the biological barriers is a challenging task, which is highlighted by the example of transdermal method of drug administration. In this paper, nonionic surfactants are primarily discussed, including their application for the fabrication of nanocarriers, their surfactant-skin interactions, the mechanisms of modulating their permeability, and the factors controlling drug encapsulation, release, and targeted delivery. Different types of nanocarriers are covered, including niosomes, transfersomes, invasomes and chitosomes, with their morphological specificity, beneficial characteristics and limitations discussed.

## 1. Introduction

An effective way to enhance the therapeutic effect of drugs is via the application of nanocontainers, which solve a variety of serious problems connected with drug toxicity and side effects, low biocompatibility and bioavailability, premature drug degradation upon storage and in biological fluids, overcoming biological barriers, etc. Currently, many different types of drug delivery systems are documented that are classified in terms of the nanocarrier material used, the administration route, the biological target, and the drug loaded [1,2,3,4,5]. In this review, special attention is devoted to amphiphilic nanocarriers, with the role of synthetic surfactants emphasized. This kind of nanocontainer is considered to be a biomimetic system due to its structural similarity with biological membranes composed of a lipid bilayer. This provides advantages for amphiphilic drug carriers in terms of biocompatibility and bioavailability over alternative delivery systems. Meanwhile, there are strong criteria towards the surfactants used for engineering the drug delivery systems, including low concentration threshold, environmentally and patient friendly properties, etc. Therefore, we focus on surfactants satisfying these criteria for delivery systems designed to be environmentally and patient friendly. Importantly, this theme closely correlates with green chemistry principles. Selected families of surfactants, their self-assembly, biodegradable properties and biomedicine potential are discussed in light of these trends.

Another important issue addressed is the overcoming of biological barriers, with aspects relevant to highlighted surfactants. Encapsulation of drugs in nanocontainers modified with cationic surfactants is reported to increase the affinity of formulations to lipid membranes of cells or organelles and bacteria walls [6,7,8,9,10], to promote blood brain barrier (BBB) penetration [11,12], thereby enhancing drug efficacy and minimizing unwanted effects. Noteworthy, the most powerful and versatile impact is connected with the enhancement of permeability due to modification of formulations with surfactants, which covers different classes of drugs, diseases and administration routes, including the transdermal, ocular, and intranasal. For this reason, one of the important areas of the review addresses the formulations enriched by absorption enhancers, especially those acting as edge activators upon transdermal drug administration. This part of the review focuses on vesicular nanocarriers of different types, such as niosomes, transfersomes, invasomes, and, supplemented by the related liposomal family, chitosomes that allow the combining of beneficial characteristics of two main classes of organic amphiphile- and polymer-based carriers.

## 2. Patient- and Eco-Friendly Amphiphilic Nanocontainers

Usage of amphiphilic compounds in various technologies, including biomedical application, attracts much attention due to their unique functional properties and capacity to form different supramolecular assemblies [9,13,14,15,16]. Of special importance is the application of surfactants as building blocks for the fabrication of nanocontainers (liposomes, micelles, niosomes, solid lipid nanoparticles, nonviral vectors, etc.) [6,7,12,17,18,19,20,21]. To enhance the therapeutic efficacy of encapsulated drugs, a variety of characteristics and factors should be taken into account, among which are the size and morphology of nanocarriers, their high loading capacity, low toxicity and side effects, prolonged circulation, cellular uptake, intracellular trafficking, targeting function, etc. Therefore, the choice of components for the design of nanocontainers is of key significance. Given the factors listed, cationic surfactants demonstrate beneficial potential in terms of their affinity toward negatively charged cell membranes, ability to integrate into the lipid bilayer, interaction with polyanions of DNA and other biological species [9,16]. At the same time, cationic surfactants are known to be rather toxic and poorly degradable [22], which limits their application. To avoid this limitation, different strategies are used, e.g., design of novel cationic surfactants with the head group differing from ammonium and bearing natural fragments, the enrichment of formulations with nontoxic nonionic surfactants, and the noncovalent modification of nontoxic lipid nanocarriers with minor additives of cationic surfactants to achieve balance between beneficial functionality and acceptable toxic effect [9,16,19,23,24].

### 2.1. Nanocontainers Based on Biodegradable Surfactants

From the viewpoint of safety of drug delivery systems, biodegradability and biocompatibility are of key importance [25]. Hence, much attention is paid to the design of surfactants satisfying these criteria. On the one hand, the degradation of drugs and carriers are unwanted processes, since significant effort is devoted to providing their stability over a long period [26]. On the other hand, formulated therapeutics should finally be susceptible to metabolic transformation and excreted from the body. This dilemma is in the focus of attention in [27], with the methods for monitoring the biological fate of drugs and nanocarriers emphasized. Noteworthy, immune system and related cells play significant roles in the degradation pathway. Coating degradation due to adsorption of plasma protein (the so-called opsonization) is assumed to be the primary mechanism of destabilizing the formulated drugs exposed to blood upon circulation, which is mediated by the recognition and accumulation of opsonized particles in mononuclear phagocyte system. The loss of therapeutic doses is further heightened by nanocarrier aggregation and loss of targeting functionality because of deactivation of targeting groups located at the periphery.

In [28], two pathways of the degradation inside the body are discussed: enzymatic hydrolysis and oxidation, which are exemplified by nonionic surfactant polysorbate 80 bearing ester groups. It was reported that enzymatic hydrolysis of polysorbate 80 can be achieved by esterases and lipases at ng/mL concentrations and phospholipase B at 200 ppm. Another pathway is the oxidative degradation of polysorbate 80, which can be accelerated by the exposure to light, the presence of peroxides, and metal ions. The degradation is shown to be promoted by the occurrence of unsaturated fragments in alkyl tails and to involve polyoxyethylene chains.

A separate substantial topic in the problem of nanocontainer degradation, which is beyond the scope of this review, is the design of stimulus-cleavable drug delivery systems [29,30]. This strategy involves the engineering of nanocarriers bearing stimuli-responsive fragments, and the cleavage is triggered by exogenous (temperature, magnetic field, ultrasound treatment, etc.) or endogenous (abnormal pH or concentration regime of the environment) factors.

Also of importance is that the majority of drugs and ingredients of nanocontainers are classified as being harmful to the environment [31]. It should be mentioned that the term “biodegradability” is primarily applicable to the environment, waste controlling, and bioremediation, and is associated with the environmentally friendly criterion. Generally, standardized microbial communities for biodegradability assays are used as inoculum that transform the testing samples via metabolic or enzymatic mechanisms [32]. Therefore, surfactant biodegradability is often considered (in analogy with any organic substance) as the ability to be assimilated by microbial communities present in water, soil and activated sludge of biological treatment facilities. There are primary and complete types of biodegradation. Primary biodegradation is structural change in surfactants caused by microorganisms, resulting in a loss of surface-active properties. Primary biodegradation forms residues that still contain useful carbon, but may be toxic and possibly more toxic than the original molecule. The subsequent decomposition of the residues formed by the primary decomposition into carbon dioxide, water, mineral salts and other low-molecular-weight components is called the ultimate (complete) biodegradation.

Reviews [33,34,35] focus on biodegradability of natural (biotic) and synthetic (abiotic) surfactants and summarize generally accepted assays. According to the Organisation for Economic Co-operation and Development (OECD) Guidelines for the Testing of Chemicals there are six standard methods for testing and ranking of readily biodegradable compounds: (i) the DOC Die-Away test, based on the evaluation of the loss of dissolved organic carbon (DOC); (ii) the CO_2_ Evolution (Modified Sturm Test); (iii) the MITI (I) (Ministry of International Trade and Industry, Japan); (iv) the Closed Bottle Test; (v) the Modified OECD Screening; and (vi) the Manometric Respirometry. Although some specificity occurs for each method, there are general guidelines, including requirements towards solubility and volatility of tested substances. The disperse sample is inoculated and incubated under specified conditions (out of air and light), normally for 28 days. Parallel, blank (inoculated without of test compounds) and reference samples containing aniline, or sodium acetate/benzoate are processed. During the assay, production of DOC, CO_2_ or oxygen is monitored to determine the degree of biodegradability. A substance may be registered as readily biodegradable if the ≥70% (DOC removal) or ≥60% (CO_2_ and O_2_ release) degradation is achieved.

It is of interest to compare the degradation of surfactants under chemical and biological factors. In [36] two geminis of the esterquat and betainate types are studied and compared. Gemini surfactants are shown to be easier to be hydrolyzed in alkaline solutions compared to monomeric analogs. In turn, the betaine-like dicationic surfactant appears to be more susceptible to hydrolytic degradation compared to the esterquat gemini. Enzymatic degradation of geminis was retarded compared to their monomeric counterparts, with no correlation observed with chemical hydrolysis. Unlike with geminis, bearing ester groups in the alkyl tails, the alternative location of cleavable fragments in the spacer is described some studies [37]. These geminis demonstrated resistance to acid hydrolysis, while being easily hydrolyzed under slightly alkaline conditions. They appeared to be more biodegradable compared to aforementioned geminis of the betainate and esterquat types. In [38], the hydrolysis of polysorbate 20 and polysorbate 80 in buffered solutions (pH 5.5, 6.5), including histidine chloride, sodium citrate, sodium succinate and sodium phosphate buffers, was studied. For the histidine buffer showing the highest rate effect, a detailed study was carried out that revealed that (i) ester hydrolysis mainly contributed to the degradation, mediated by imidazolium residue of histidine; (ii) oxidation and aminolysis were minor processes; polysorbate 80 showed easier degradation compared to polysorbate 20; (iv) the presence of unsaturated bonds promoted the degradation, thereby indicating that all-oleate polysorbates are not beneficial components for nanocontainers; (v) the formulation admixed with monoclonal antibodies may protect surfactants against histidine catalyzed degradation.

An unusual temperature effect on alkaline hydrolysis of nonionic geminis has been demonstrated in some studies [39,40]. A decrease of reaction rate with an increase in temperature was observed both for geminis with carbonate bonds in the spacer and their monomeric analogs. The authors interpreted this result in terms of the reverse solubility-temperature behavior of the surfactants. In addition, hydrophilicity and resistance toward hydrolytic degradation of different weak bonds (ester, amide and carbonate) are compared [39]. Carbonate-bearing surfactants are reported to be more stable against alkali hydrolysis compared to those with an ester fragment.

The type, structure and physicochemical properties of amphiphiles play an important role in biodegradation. An increase of the alkyl chain length typically results in the decrease in the biodegradation rate of surfactants due to a decreased in the compound solubility in the homologous series. Another reason for low biodegradability may be associated with surfactant antimicrobial activity, which depends on the alkyl chain length.

Nonionic surfactants have better biodegradability than other surfactant types. However, their degradation products may be toxic to the environment. For example, the biodegradation of oxyethylated alkyl phenols, which are the part of nonionic surfactants, occurs through gradual shortening of the ethylene oxide chain and carboxylation of the alkyl tail. The highly stable mono- and diethoxylates of 4-alkylphenol are formed with partial hydrolysis of the oxyethylene chain. These compounds are poorly soluble in water, toxic, and only 50% of these detergents are destroyed [41].

Anionic surfactants are also biodegradable. Linear alkylbenzenesulfonates undergo enzymatic aerobic degradation by microorganisms from 97% to 99%. The high degree of decomposition of linear alkylbenzenesulfonates is related with their molecular structure; the farther the position of the benzene ring from the central carbon atom, the easier its destruction [34].

Cationic surfactants have low biodegradability and undergo only aerobic oxidation. The almost complete absence of oxygen in the sludge leads to the fact that the toxicity of cationic surfactants persists for a long time. Cationic surfactants have a strong bactericidal activity. They are easily adsorbed in sediments, minerals or organic substances in wastewater, which also hinders their biodegradation. However, due to their high surface activity, low critical micelle concentration (CMC), and significant solubilization capacity, they find application in various fields of industry. One of the advantages of cationic surfactants is the possibility of chemical modifications of their scaffold, including the introduction of fragments that meet biological criteria [9,42,43].

The best known and commercially available example of degradable surfactants is quaternary ammonium compounds with ester groups [44]. Biodegradation of quaternary ammonium compounds depends on the molecular structure, concentration and their resistance against microorganisms. It is believed that microorganisms metabolize quaternary ammonium compounds under aerobic conditions according to three mechanisms. In the first case, the alkyl chain is hydroxylated at the terminal carbon atom, followed by oxidation to the aldehyde group. Furthermore, the compounds undergo β-oxidation with the formation of acetyl-CoA, which is included in the citric acid cycle. The second mechanism involves α-hydroxylation of the alkyl fragment, followed by the cleavage of the C–N bond under the enzymes’ action. Complete decomposition terminates by β-oxidation. The third mechanism, in addition to the hydroxylation of long chain alkyl, involves hydroxylation and demethylation with the formation of alcohol and alkylamine [45].

Natural surfactants derived from plant or animal sources are highly biodegradable compared to synthetic surfactants [46]. However, it should be noted that the extraction of natural surfactants is very expensive. In this case, synthetic surfactants containing ester, amide, disulfide or other degradable moieties are a good alternative [47,48,49,50].

### 2.2. Biodegradable Surfactants

#### 2.2.1. Amino Acid Surfactants

Amino acid-based surfactants, which can be degraded by enzymes into non-toxic fragments, are attracting more and more attention [51,52,53,54]. For example, arginine-based surfactants demonstrate a good antimicrobial activity, biodegradability, and low toxicity [55]. In [56] the lysine-based surfactants (hydrochloride salts of Nε- and Nα-acyl lysine methyl ester) were studied as potential agents for inclusion in nanocarriers. The membrane-destructive activity of surfactants was studied using erythrocytes as a model of the endosomal membrane. Only compounds with a positive charge on the α-amino group of lysine showed pH-sensitive hemolytic activity. Moreover, the ability to destroy cell membranes decreases with an increase in the alkyl chain length from 14 to 16 carbon atoms. In addition, the hemolysis data showed that both amphiphiles can achieve maximum membranotropic activity in late endosomes. Improved hemolytic kinetics allows disruption of endosomal membranes before transformation of endosomes to lysosomes. Similar results were obtained for chitosan-tripolyphosphate nanoparticles [57]. Nanocontainers were developed using two pH-sensitive surfactants based on amino acids of the Nα, Nε-dioctanoyl lysine series. In particular, at pH 7.4, a slight membrane permeability was observed. At acidic pH, prevailing in endosomes, membrane-destabilizing activity was shown. In [58], the interaction mechanism of gemini surfactants based on lysine with model biomembranes was studied. It was found that for effective interaction of surfactants with phospholipid vesicles and their further destabilization, it is necessary to maintain the optimal balance of hydrophobic, electrostatic interactions and hydrogen bonding. From this viewpoint, the optimal system turned out to be a surfactant with four lysine fragments in a spacer (12-(Lys)_4_-12), since it loosened lipid membranes, which led to their destruction.

**Figure 1:**
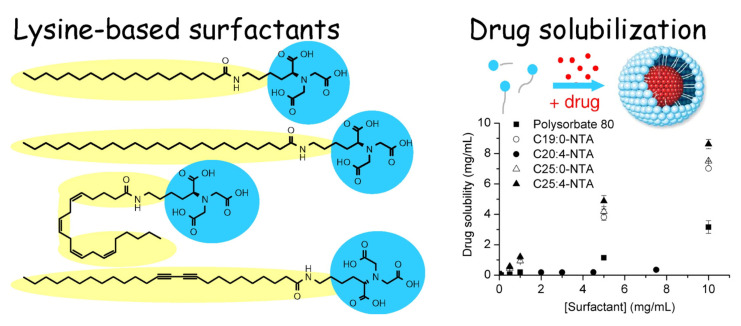
Lysine-based surfactants for the solubilization of anticancer drugs [59]. Copyright 2012 Elsevier.

Nanocontainers based on amino acid surfactants can be used as carriers for a wide range of medicines [22,34,41,42,43,44,45,46,47,48,49,50,51,52,53,54]. Lysine-based surfactants were obtained to improve the solubilization of water insoluble anticancer drugs [59]. It was found that these surfactants increased the drug solubility from 0.15 μg/mL to 7 mg/mL with an encapsulation efficiency (EE%) of 46%. In addition, the nanocontainers had insignificant cytotoxicity (Figure 1).

**Figure 2:**
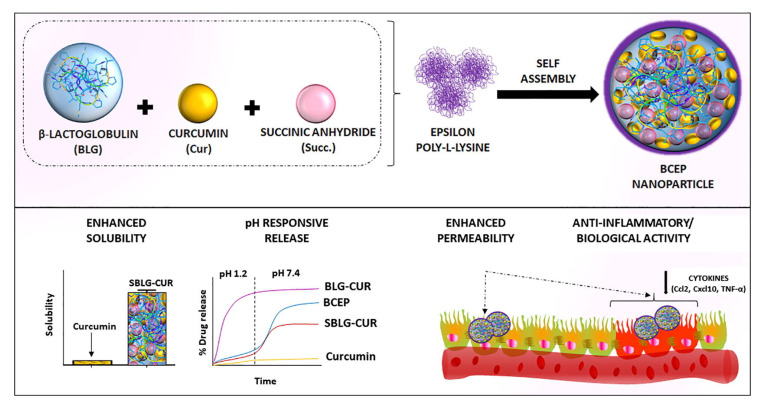
A novel pH responsive nanocolloids based on bovine β-lactoglobulin and succinylated β-lactoglobulin cross-linked with epsilon poly-l-lysine [61]. Copyright 2021 Elsevier.

The formulation combining a cationic surfactant based on lysine (Nε-myristoyl lysine methyl ester) and sodium hyaluronic acid was developed for viscose tissue treatment as a wound healing coating [60]. Viscose samples treated in this way showed pronounced antimicrobial property when tested on gram-positive and gram-negative bacteria, and some pathogenic fungi. The authors of [61] managed to obtain nanocontainers based on lysine derivatives for oral use. They were stable in the acidic environment of the stomach and demonstrated prolonged drug release (curcumin) under conditions simulating the small intestine (Figure 2). This dosage form of curcumin reduced the production of inflammatory cytokines and chemokines such as TNFa and CXCL10 more than curcumin solution or suspension.

**Figure 3:**
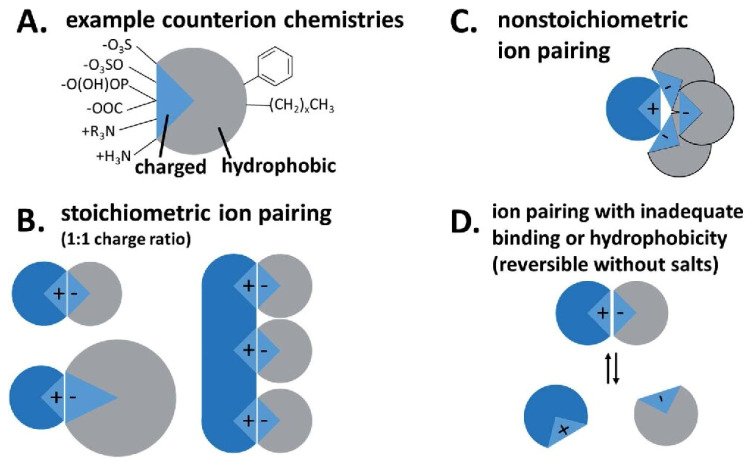
Scheme of hydrophobic ion pairing. (**A**) Possible charged groups (left) and hydrophobic moieties (right) for a counterion. (**B**) Stoichiometric ion pairing between a cationic active pharmaceutical ingredient (blue) and anionic counterion. (**C**) Non-stoichiometric ion pairing. (**D**) Reversible ion pairing due to inadequate binding or hydrophobicity [62].

It is also interesting to use amino acid surfactants as components to create hydrophobic ion pairs (HIP) (Figure 3). In the case of using surfactants as counterions, the drug hydrophobicity can be significantly increased by masking the drug charge with lipophilic surfactant tails [62]. This promotes the inclusion of drug molecules in surfactant-based nanocontainers, which can improve their oral bioavailability [63,64].

**Figure 4:**
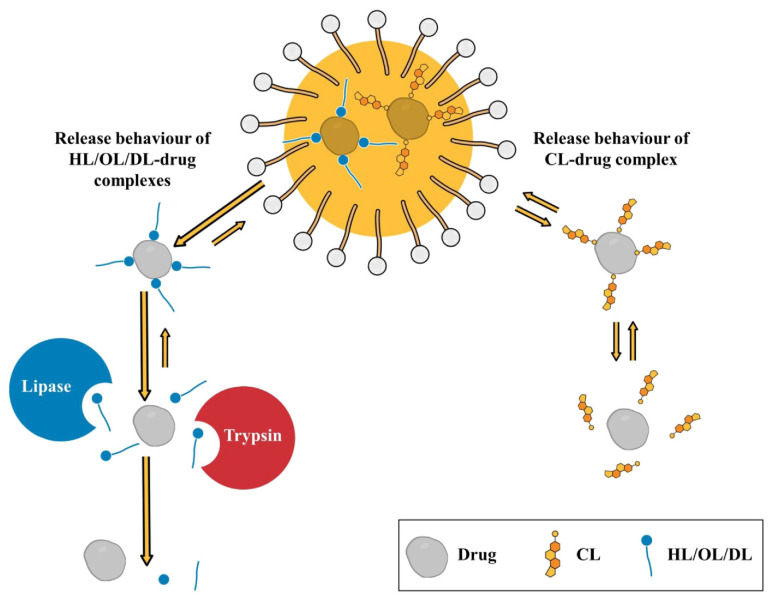
Anticipated release behavior of hexadecyl lysinate (HL), oleyl lysinate (OL) and decyl lysinate (DL)-drug complexes in comparison to the release of cholesteryl lysinate (CL) complexes out of lipid-based nanocarrier systems [51].

In [51], the degradation of lysine-based surfactants was studied. Enzymatic cleavage of cholesterol lysinate (CL), oleyl lysinate (OL) and decyl lysinate (DL) was investigated by incubating lysine esters with isolated enzymes. The rapid degradation by trypsin and lipase was observed for DL and OL. DL was cleaved by both enzymes with residual amounts of <1% after 5 min, while trypsin and lipase completely cleaved OL after 30 and 45 min, respectively. No significant degradation was observed for CL during 24 h by trypsin, lipase, or cholesterol esterase. In contrast, degradation studies carried out on the pig’s intestinal mucosa showed cleavage of CL by brush border enzymes. After 30 min, only 17.6% of CL was determined in the test solution. According to these results, CL, OL and DL can be degraded into non-toxic products when taken orally, preventing side effects (Figure 4).

Hexadecyl lysinate improved the effectiveness of nanocontainers for DNA [52]. The formation of a hydrophobic ion pair made it possible to obtain an efficient non-viral vector, which is capable of transfection on HEK-293 cells. In addition, enzymatic degradation studies showed the rapid destruction of hexadecyl lysinate by isolated enzymes and Caco-2 cells, and experiments on cell viability did not reveal the toxic effect of the surfactant.

Also, complexes with drugs by the HIP mechanism were obtained for arginine surfactants with an ester fragment that can be cleaved in vivo by endogenous enzymes [53]. The arginine surfactants with low cytotoxicity were rapidly degraded by trypsin. They also effectively formed HIPs with the hydrophilic anionic macromolecular drugs heparin and daptomycin. In [65], the vesicular systems based on biocompatible diacyl glycerol-arginine surfactants were obtained. These aggregates were loaded with ciprofloxacin and 5-fluorouracil. It was shown that the percentage of encapsulated drug depends on the physicochemical properties of the carrier and the type of drug. Antimicrobial activity of empty and ciprofloxacin-loaded vesicles was assessed against gram-positive and gram-negative bacteria (*Escherichia coli*, *Staphylococcus aureus* and *Klebsiella pneumoniae*). It was found that the nanocontainers themselves have antimicrobial activity. Vesicles loaded with ciprofloxacin showed similar or higher antimicrobial activity than free drug solution [65,66].

The arginine surfactants are also used for modification of liposomal systems [67]. Liposomes were loaded with the new potential anticancer substances—2-benzimidazolylquinoxalines. Arginine liposomes loaded with 2-benzimidazolylquinoxalines showed efficient cellular uptake and a more pronounced cytotoxic effect toward the HeLa cancer cell line and were less toxic toward the normal Chang liver cell line. Liposomal formulations with an arginine surfactant (N-lauroyl amide dihydrochloride) and blood protein transferrin were developed as non-viral vectors [68]. The transfection efficiency of these systems was directly related to the presence of a non-toxic arginine surfactant in lipid composition. Better transfection profiles were found for lipoplexes with surfactant inclusion. This action is due to the fact that lipids, surfactants and transferrin act synergistically, promoting the escape of DNA complexes from endosomes, thus improving transfection compared to systems without surfactants. The mechanism of arginine surfactant action on the membranes of human red blood cells was discussed in one study [69], which reported that the destruction of erythrocyte membranes proceeded in two ways. At low surfactant concentrations, the incorporation of surfactant monomers into the erythrocyte membrane with subsequent detachment of microvesicles was responsible for hemolysis. At high surfactant concentrations, it is a micellar mechanism that causes erythrocyte membrane solubilization and further cell lysis.

Amino acid surfactants can act as solubilizing agents for antifungal drugs. Amphotericin B, due to its poor solubility, is sold as a colloidal suspension based on simple bile salt sodium deoxycholate (used as a solubilizing agent). However, this formulation has serious side effects. It was found that *N*,*N*’-bis(octylcarbamoyl) gemini, derived from cysteine, forms aggregates at lower concentrations than bile salt under physiological conditions. The resulting aggregates showed better solubilizing activity toward Amphotericin B [70,71]. Micelles of gemini surfactants solubilized Amphotericin B in monomeric form, which reduced its toxicity. At the same time, as can be seen from the minimum inhibitory concentration (MIC) values, the effectiveness of Amphotericin B action also slightly decreased. For another surfactant with a cysteine fragment, an equimolar mixture with bile salts of sodium cholate and sodium deoxycholoate was prepared [72]. The resulting nanocontainers were also assessed for Amphotericin B delivery. The results showed that mixed micellar systems improve drug solubilization (in its monomeric and less toxic form) and exhibit in vitro antifungal activity against *Candida albicans* compared to a commercial formulation. These surfactants are promising delivery systems due to the possibility of the disulfide bond reductive cleavage to control drug release from micelles.

#### 2.2.2. Surfactants with Ester and Carbamate Fragments

Much attention is paid to surfactants that contain amide, carbamate, ether and ester groups [73]. A number of studies proved that surfactants containing amides or esters have excellent biodegradability and can undergo enzymatic hydrolysis to non-toxic fatty acids, choline and other substances [74]. Some publications focused on surfactant-based nanocontainers with ether and ester moieties. The authors of [75] proposed micellar aggregates based on gemini surfactants with ester fragments as tissue softeners due to their ability to effectively solubilize organic substances. The ability of this type of surfactant to act as nanocontainers for hydrophobic substrates was evaluated in [75,76]. An anthraquinone dye, quinizarin, and an azo dye, Sudan I, were chosen as model substrates. It was found that the introduction of ester fragments into the alkyl chains leads to a decrease in the solubilization ability of surfactants compared with non-functionalized gemini surfactants [76]. In addition, the possibility of creating UV-sensitive nanocontainers was revealed [9].

Betaine esters are a special class with a pH dependence. These compounds are hydrolyzed with the formation of two harmless products—the amino acid betaine and long-chain alcohol [77]. The authors of [48] synthesized cationic oligomeric betaine surfactants, which demonstrated a high percentage of biodegradation compared with dicationic surfactants without ester groups. However, despite a significant increase of the biodegradation rate caused by the presence of labile ester bonds, these compounds are not totally degradable (<60%). Incomplete degradation of these oligomeric surfactants is explained by the resistance to biodegradation of the hydrophilic cationic part formed during the hydrolysis of the ester bond by microorganisms. It should be noted that these compounds do not prevent the decomposition of readily biodegradable organic compounds by activated sludge, and, therefore, their presence in wastewater should not affect the functioning of the biological treatment process. The studied acute toxicity of betainate oligomeric surfactants on Daphnia magna increased with the lengthening of alkyl chains, which suggests that the hydrophobicity of the molecule determines the aqueous toxicity of these surfactants.

It was shown that the presence of carbonate linkages in the structure of gemini surfactants both in the spacer and in the hydrophobic tail accelerates biodegradation [78,79]. They can be enzymatically hydrolyzed by microbes with the release of carbon dioxide and the formation of readily biodegradable quaternary ammonium alcohols.

A number of publications are devoted to the study of cationic surfactants with a carbamate fragment and nanocontainers based on them. Carbamate amphiphiles were tested as solubilizing agents for hydrophobic probes and drugs [8,23,43,80,81,82,83]. In particular, it was found that the presence of carbamate fragments of different hydrophobicity in the structure of ammonium surfactants makes it possible to obtain aggregates of varying morphology. This leads to an increased solubilization ability of nanocontainers toward the model dye Orange OT and the anti-inflammatory drugs indomethacin and meloxicam [81]. A hexadecyl derivative with a carbamate fragment was also investigated in a mixture with a nonionic surfactant. Mixed systems demonstrated effective solubilization of the anti-inflammatory drug meloxicam. Moreover, a balance between low toxicity and high solubilizing activity was achieved [23]. Different cationic surfactants were used for creation of nanocontainers for the Lontrel^®^ herbicide [82]. The use of carbamate-bearing surfactants made it possible to increase the herbicide concentration in plants threefold. Improved wetting ability was also achieved.

**Figure 5:**
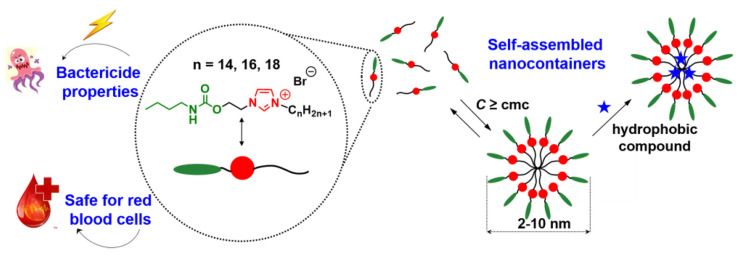
Some properties of carbamate surfactants with an imidazolium head group [8]. Copyright 2020 Elsevier.

Carbamate surfactants with an imidazolium head group are promising agents for biomedical applications (Figure 5) [8,43,83].

They demonstrated a high solubilizing ability toward hydrophobic substrates, antimicrobial properties, low hemolytic activity, and the ability to bind with the DNA decamer and protein. In particular, it was found that the interaction of amphiphiles with the DNA decamer is predominantly cooperative. However, in the case of hexadecyl and octadecyl derivatives, the intercalation of surfactants between the nitrogenous bases of the nucleotide occurs also at the molecular level, while non-functionalized imidazolium surfactants interacted with the DNA decamer only due to the intercalation mechanism [84,85,86]. For surfactant/protein complexes, it was revealed that the binding of components occurs predominantly at the tryptophan amino acid residue due to various types of non-covalent interactions. Carbamate surfactants unfold a small part of protein molecules; nevertheless, the effect of surfactants on protein is reversible. In addition, surfactant/BSA complexes had a more pronounced solubilization effect in contrast to individual surfactant micelles.

**Figure 6:**
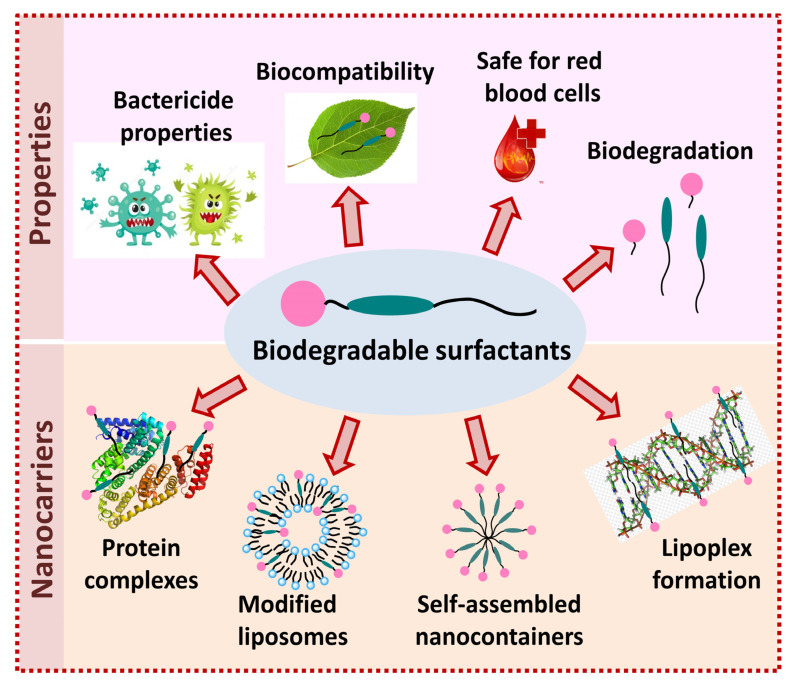
Properties of biodegradable surfactants and nanocarriers based on them.

Thus, biodegradable surfactants can be used as building blocks for micellar nanocontainers or complexes with biopolymers, and act as surface modifiers of lipid carriers (Figure 6). The range of substrates that can be bound by nanocarriers is quite extensive and determined by the type of nanocontainer. This leads to an improvement of the cytotoxic properties of encapsulated drugs toward pathological cells, an increase in the selectivity index and in the ability to escape endosomes.

To complete the topic of micellar nanocontainers, some key points need to be emphasized. An important property of micelles is their ability to increase the solubility and bioavailability of poorly soluble drugs [87,88,89]. In addition, micellar systems have the advantage of spontaneous formation, which only requires maintaining CMC [90]. Because of their small size, micelles show spontaneous penetration into organs and tissues due to the enhanced permeability and retention effect. However, micelles are dynamic systems that do not have a constant composition and are not stable in biological media [91]. This leads to the collapse of micelles in the blood immediately after administration in vivo. The process is accompanied by the subsequent deposition of the incorporated drug or its transfer into plasma proteins due to the instant dilution of the surfactant solution by blood [92]. Therefore, in the development of nanocontainers for drug delivery, there is a need to modify micellar nanocontainers or to find alternative types of carriers. Polymer micelles [93] and liposomal systems [94] can be a good alternative from this point of view. An additional way to solve the problem is to decorate micelles with a more stable organic shell, e.g., polyelectrolyte layer. This approach has been used in the layer-by-layer protocol, in which a micellar core composed of cationic surfactants was used as a template for the deposition of polyanion/polycation pairs. In this case, multiple tasks can be achieved: (i) hydrophobic guest solubilization; (ii) stabilization of a micellar nanocontainer loaded with drug or probe; (iii) the electrostatic promotion of adsorption of the first oppositely charged polyelectrolyte layer [95,96,97,98].

## 3. Nanoencapsulation of Drugs as Powerful and Versatile Tool to Cross the Biological Barriers

Biological barriers are essential for the functioning of many human organs and are responsible for protecting the organism from physical, chemical and biological damage. The barrier functions of the cell membrane play a key role in maintaining homeostasis, ensuring a stable behavior of biological processes. Every living organism is in a state of constant exchange with the external environment. In addition, the cells of multicellular organisms are in a state of exchange with the intercellular environment. In this context, there are two types of biological barriers: the external barriers between organism and habitat and the barriers within the organism that provide homeostasis of individual organs. In humans, external barriers include the skin, respiratory and digestive systems. Internal barrier functions are implemented mainly due to the structural features of the capillaries that supply blood to organs and tissues. This allows for the maintenance of differences between internal fluid in the organ compared to the composition of the blood. Examples of internal barriers are blood-brain, blood-ocular, blood-testis, blood–bile, placental and other biological barriers [99,100,101,102,103,104,105].

Consequently, foreign substances including drugs are forced to cross these barriers to reach the target site, which is a challenging task in the field of drug delivery. One of the effective ways for overcoming the biological barriers is the application of nanocarriers. In the scope of this review, priority is given to formulations involving amphiphilic building blocks with surfactant-based vesicles emphasized. For the latter, a marked enhancement of permeability is documented [106,107,108], which presents them as beneficial nanocarriers for transdermal delivery of therapeutics. Therefore, in this review, we introduce transdermal drug delivery nanosystems as those showing advantages over other routes as being non-invasive, avoiding first-pass hepatic metabolism and providing continuous drug administration [109].

Skin is a main part of the integumentary system that is the biggest multilayer interface between our body and the outside world. The integumentary system includes the skin, hypodermis, associated glands, hair and nails [110]. The skin is made up of two layers: the superficial epidermis and the inner subjacent dermis. The epidermis is the tough outer layer that acts as the first line of defense against the external environment. From superficial to deep, the primary layer is the 10–20 µm thick stratum corneum (SC); this layer forms the first defense against drug delivery systems. It is composed of densely packed and highly keratinized dead cells. Neath SC epidermis has four layers: (1) stratum lucidum; (2) stratum granulosum; (3) stratum spinosum; and (4) basal cell layer. It is important to note that in the stratum granulosum, cells are connected via tight junctions that form an additional barrier for the transdermal drug delivery systems.

The epidermis is separated from the dermis by a 0.2–0.3 µm thick basement lamina that has its own barrier functions, because forms felt-like a layer that acts as a filter between epidermis and derma. This filter limits the penetration of molecules with a weight of >40 kDa. Importantly, the epidermis itself is devoid of blood supply and gets nutrition from the underlying dermis, which contains a network of blood vessels, nerves, sweat glands and hair follicles [111]. In general, the aim of a transdermal delivery system is to pass via epidermis (mainly SC), reach the capillaries of dermis and then the systemic blood circulation. The hypodermis is the layer between dermis and underlying organs. This layer provides additional cushion and insulation through its fat storage function and connects the skin to underlying structures such as muscle [110].

It was shown that there are three main pathways for penetration of nanosystems through the epidermis [112].

(1) It is assumed that the intercellular pathway plays a major role in skin permeability for the particles with a size > 10 nm [113].

(2) The transcellular pathway involves cellular uptake of the delivery system by keratinocytes. This pathway is very specific, because the delivery system has to overcome the multilayer skin structure [113].

(3) The trans-appendageal pathway involves sweat and sebaceous glands or hair follicles and is a common route for delivery of the systems with a size of 20–40 nm [114]. The trans-appendageal pathway can be considered as the least significant, because the appendages cover only 0.1% of the skin surface [115]. On the other hand, it is the only pathway for the large particles [112].

With transdermal administration, the medicines, as with intravenous injection, enter the systemic bloodstream immediately, without adverse effects on the mucosa of the gastrointestinal tract. The simplicity and painlessness, safety, absence of the need for frequent intake, and the possibility to prescribe this form to patients who have difficulties in chewing and swallowing are undoubted advantages of this drug administration route [116,117,118]. The main requirement for medicines selected for transdermal administration is a small molecular weight, and it should not exceed 500 Da. To overcome the skin barrier, the molecule also must have certain physicochemical properties: (1) good permeability through the skin due to amphiphilic properties; (2) no irritant effect on the skin; and (3) the possibility of use in low doses. All of these parameters affect the degree of drug penetration through the skin.

Passage through intact skin is difficult for most medicines. It occurs for two main reasons: the gradient of substance concentration and the water gradient. Achieving a substance gradient is more difficult because it requires a balance between the lowest concentration needed and the highest concentration that is limited by the toxicity and own solubility of substance. Therefore, the penetration of lipids through the skin is mainly the result of a transdermal hydration gradient. The lipid carrier on the skin surface resists dehydration and is absorbed into deeper layers. It is better not to use occlusive applications, since an osmotic gradient occurs between the SC and dermis in dehydration conditions [119].

In [106] the optimized parameters are categorized that allow for the improving of the efficacy of formulations: (i) skin hydration that increases percutaneous absorption and can be contributed by occlusive effect preventing water loss from the skin; (ii) mixing/fusion effect due to structural similarity of nanocarriers composed of amphiphilic molecules with lipids of SC; (iii) deformability of formulations; (iv) disrupting effect of nanocarriers toward the SC, which results in solubilizing the component of SC and fluidize its structure; and (v) size and charge characteristics, which is the most debatable point, since there are disparate viewpoints on the charge of nanocarriers enhancing the skin penetration. Surfactants demonstrate versatile mechanisms of modulating the permeability, including stabilization of formulations and the optimization of their size and stability. Their action can be also considered in correlation with the reduction of interfacial tension, which promotes the skin-nanocarrier contact. Due to solubilization activity, the surfactants enhance the concentration of drugs and their therapeutic efficacy.

The skin-surfactant interaction is a subject of discussion in [120]. Among others, the problem of surfactant irritation potential is analyzed. This is mainly associated with the surfactant interaction with proteins in the SC, which can result in their denaturation and swelling. It was concluded that the responsibility for the skin irritation effect lies firstly with the monomeric surfactant. For this reason, gemini surfactants are reported to show less irritant activity. Surfactant interactions with the SC’s keratin proteins are successfully modeled by surfactant-BSA complexation due to the structural similarity of both proteins. Different from surfactant-protein interactions, surfactant interaction with lipid components of SC is studied less from the viewpoint of irritation potential. It was postulated that surfactant monomers can integrate with the lipid bilayer of SC, thereby disturbing its structure and increasing permeability. Aggregated surfactants probably show a solubilization effect toward SC lipids, which can induce skin dryness. These factors should be taken into account upon the practical application of surfactants in cosmetics and biomedicine. In particular, they can be controlled by the proper choice of the surfactant structure, concentration and the optimization of formulations.

### 3.1. Lipid-Based Nanocarriers

**Figure 7:**
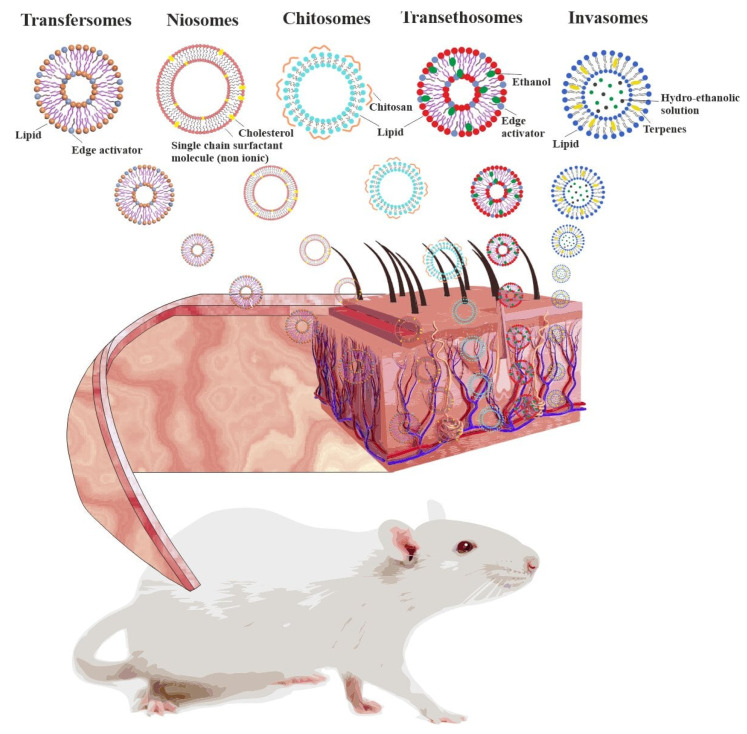
Different types of lipid-based nanocarriers used in transdermal drug delivery.

The use of vesicular systems based on surfactants, including niosomes, transfersomes, transethosomes, chitosomes, invasomes (Figure 7), for transdermal drug delivery will be discussed in detail below. In fact, vesicular nanoparticles are the most widely used drug delivery vehicles. Maintaining a constant medicine concentration for a relatively long time and protecting it from the enzymes hydrolytic action is ensured by the gradual drug release from the nanocontainer [12,121,122,123]. The structure of vesicular-type carriers allows encapsulation of both hydrophilic and hydrophobic molecules. In this case, hydrophilic substrates are located in the internal water space, while lipophilic—inside the bilayer. There are different ways of liposomal drug application. In order to overcome the known disadvantages of invasive methods, non-invasive methods are proposed, such as oral, buccal, pulmonary, nasal, and transdermal.

#### 3.1.1. Niosomes

Niosomes are microscopic vesicles consisting of an aqueous core surrounded by a membrane of nonionic surfactants, which form closed two-layer structures. Cholesterol or its derivatives are often used as excipients, which increase the bilayer stability and influence its fluidity and permeability [124,125]. This helps to protect the medicines from degradation or inactivation. Niosomes have several advantages as a delivery system: long lifetime, simple preparation and modification, chemical indifference, high biocompatibility, low toxicity and biodegradability [126,127,128]. A tendency to aggregation and partial loss of the encapsulated drug from the nanocontainer during delivery are the main disadvantages of niosomes.

Niosomes were obtained for the first time by the French personal care company L′Oreal in the 1970s. Subsequently, they began to be studied as drug delivery systems. In the last two years alone, more than 200 articles on niosomes were published, 20% of which are in the form of reviews. Some of the papers on niosomes released for 2019–2021 are set out in Table 1.

Niosomes, loaded with pharmaceutical substances, can be administered via different ways depending on the disease, prescription and composition of the drug [144]. The transdermal route is highlighted among the various methods of drug administration [145]. There are reports that niosomes can penetrate deep into the skin [146,147] and deliver drugs into the systemic bloodstream [148]. Niosomal systems can interact with the SC by aggregation, adhesion and fusion, which results in a high thermodynamic activity gradient of the drug at the vesicle-skin interface. Thus, it creates a driving force for drug penetration [147,149]. A comparison of submicelles and niosomes loaded with a hydrophilic antimicrobial drug (sulfadiazine sodium salt) was carried out to increase drug permeability through the skin [150]. The ex vivo experiments showed that only niosomes act as effective agents for transdermal delivery. In addition, the review observes that surfactants belonging to the Pluronic class are capable of spontaneously forming vesicles and can be used as permeability enhancers. It should be noted that the shape, size, elasticity, surface charge, and viscosity of niosomes could influence the permeability. These parameters can be modulated by the type of surfactant, the amount of cholesterol, the method of niosomes preparation, and the addition of charged molecules or polymers [151].

Polyoxyethylene ethers (Brij), sorbitan esters (Span), and polyoxyethylene sorbitans (Tween) are the most often used nonionic surfactants for the preparation of niosomes. Their combination can be used to increase the flexibility and stability of nanoparticles [152]. Surfactants improve drug permeability through SC in two ways. First, due to their ability to reduce surface tension, they increase fluidity and solubilize and extract lipids from the epidermis. They can also lead to corneocyte disruption through binding and interaction with keratin filaments [153]. Secondly, the adhesion of niosomes to the skin surface causes changes of the SC properties by reducing water loss from the epidermis. It leads to an increase of skin hydration and to loosening of the tightly packed cell structure [147].

Despite all the advantages of niosomes as delivery systems, they do not have the necessary rheological properties to be used for topical application. The inclusion of gels capable of increasing viscosity of niosomal systems could be a solution of this problem. Viscosity can be a determining factor for drug retention and penetration, since more viscous gels act as a second barrier, protecting them and facilitating prolonged release. Polymeric hydrogels based on carbopols, poloxamers, chitosan, hyaluronic acid, etc., which, having high biocompatibility with the skin, are mainly used to impart viscosity to the drug delivery system [151]. Inclusion of polymers in the vesicle can improve the stability and physicochemical properties of niosomes [154,155].

Literature data analysis showed that a large number of publications are devoted to niosomal gels that have been successfully tested on animals. For example, a niosomal gel containing febuxostat was successfully tested on rabbits [149]. The gel composition showed maximum anti-gout efficacy compared with standard (pure) febuxostat, while demonstrating high stability during storage in different temperature ranges. The authors of [156] successfully encapsulated the hydrophilic drug arbutin in niosomes. They showed that the gel-like niosomes with arbutin were the most permeable compared with the conventional gel and showed no toxicity on the healthy human foreskin fibroblast cells. Hyaluronan containing niosomes were used to coencapsulate curcumin and quercetin and showed higher antioxidant and anti-inflammatory effects compared with unmodified niosomes [154]. It was shown that the gel-like niosomal composition loaded with lacidipine was more than twofold more permeable than the control gel [157]. The study of antihypertensive activity confirmed the efficacy of the transdermal delivery of lacidipine. The reduction of the arterial pressure of rats using the niosomal gel was gradual rather than abrupt, and this was also observed in the case of oral administration, which allowed for the maintenance of normal blood pressure for 48 h. Histopathological examination revealed no irritant effect on the skin.

Another way of improving the stability and storage time of niosomes is introducing a certain amount of cationic additives into their structure to obtain a positively charged system [158,159]. Among the methods for imparting a positive charge, the incorporation of cationic surfactants into niosomes is the most often used pathway [160,161,162]. This may increase the stability of composition and may affect the loading efficiency and the release rate of bioactive compounds [163]. Thus, niosomes are a fairly new and efficient drug delivery system. Niosomes are more stable and cheaper than other vesicular systems. They are becoming promising carriers for controlling drug release rates, targeting drugs into the specific tissues or cells, reducing systemic side effects, and toxicity. Research in the field of niosomes has expanded, which could lead to the developing of efficient dosage forms in the pharmaceutical industry.

#### 3.1.2. Transfersomes and Transethosomes

**Figure 8:**
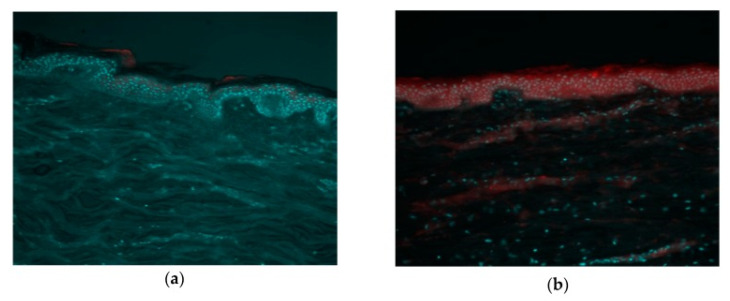
Fluorescence microscopy images of a pig-ear skin cross-section. Red color corresponds to Nile red fluorescence and blue to Hoeschst staining of the cell nucleus. (**a**) Not vehiculized Nile red control (image J, mean epidermis intensity 7846 ± 140 AU); (**b**) Nile red-marked transfersomes (image J, mean epidermis intensity 12,428 ± 254 AU). The images were captured using 10× magnifications [165].

Transfersomes are special lipid aggregates that can penetrate through pores of a much smaller size than their own, which is due to their extremely high deformability [164]. Transfersomes were originally observed by Cevc and Blume in 1992 as delivery systems consisting of two components which significantly differ from each other in their stabilizing (lipid)/destabilizing (edge activator) properties [119]. The presence of an edge activator in the composition of transfersomes is believed to increase their deformability by decreasing their interfacial tension. They are able to squeeze themselves through intercellular regions of the SC and prefer tortuous intercellular pathways of penetration. In order to confirm the penetration of transfersomes deep into the skin, it is most convenient to use fluorescence microscopy. As can be seen in Figure 8, the fluorescence of the free Nile red is observed only in SC, while the transfersomal Nile red penetrates deep into the skin. However, the penetration mechanism of transfersomes through the skin are still the subject of study and discussion.

**Figure 9:**
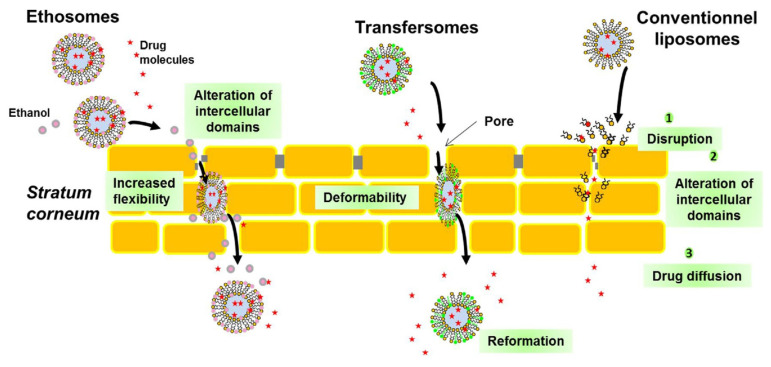
The main permeation mechanisms of different lipid-based nanovehicles [168]. Copyright 2018 Elsevier.

In 2000, eight years after the discovery of transfersomes, ethosomes containing a relatively high percentage of ethanol were proposed to overcome the skin barrier [166]. The ethosomes may increase drug penetration due to the fact that ethanol increases the substrate’s solubility and the fluidity of SC lipids (Figure 9). The combination of two delivery systems in one with increased penetration through skin barriers—transfersomes and ethosomes—led to the production of transethosomes, which were first offered in 2012 by Song et al. [167].

Recently, a large number of reviews have been published on the topic of transfersomal systems, in which the success of their use has been demonstrated [122,169,170,171,172,173,174,175]. Phospholipone 90 G [176], egg phosphatidylcholine [177,178], soybean phosphatidylcholine [179] and nonionic surfactants Tween and Span are most commonly used as components of transfersomes. Depending on the target or on the encapsulated substance, the composition can be modified, and this directly affects the transfersome properties. The easiest way to change the transfersome properties is to vary the ratio of components and type of surfactant [153]. For example, 12 systems with different surfactants, drug: phosphatidylcholine and surfactant:phosphatidylcholine ratios were studied in [180]. Adding sodium lauryl sulphate to the system led to an increase of EE% of the hydrophilic drug ivabradine hydrochloride compared to cases with Tween 80 and cetrimide. The authors explain this phenomenon in terms of different HLBs, hydrocarbon tail length, and the structural and physicochemical characteristics of the surfactants studied. It is possible to achieve the necessary size, charge and EE% by varying the ratio of components. In [181], three phospholipid:surfactant ratios were varied (90:10, 85:15, and 75:25) and, according to the results, a components ratio of 85:15 proved to be optimal in all parameters, while in [180] the system with ratio of 75:25 had the best size and charge as well as high EE% values and prolonged substrate release.

Currently, researchers have moved far away from the classical transfersome composition. Along with deformability, the increase of effectiveness of nanoparticles is very interesting. In addition to classical surfactants, there are works on the formation of transfersomes with the inclusion of not quite classical surfactants. In [182], succinate D-α-tocopheryl polyethylene glycol 1000 (TPGS), formed by esterification of vitamin E with polyethylene glycol 1000, acted as the edge activator. The system was modified with folic acid to achieve selectivity of the systems toward tumor cells. In this case, TPGS not only plays the role of an edge activator, but also acts as a stabilizer to avoid the removal by reticuloendothelial system and helps to improve membrane permeability. The authors of [183] demonstrated mannosylated naringenin-loaded transfersomes, which can effectively target cancer cell macrophages by specifically binding with mannose ligands expressed in immune system cells.

**Figure 10:**
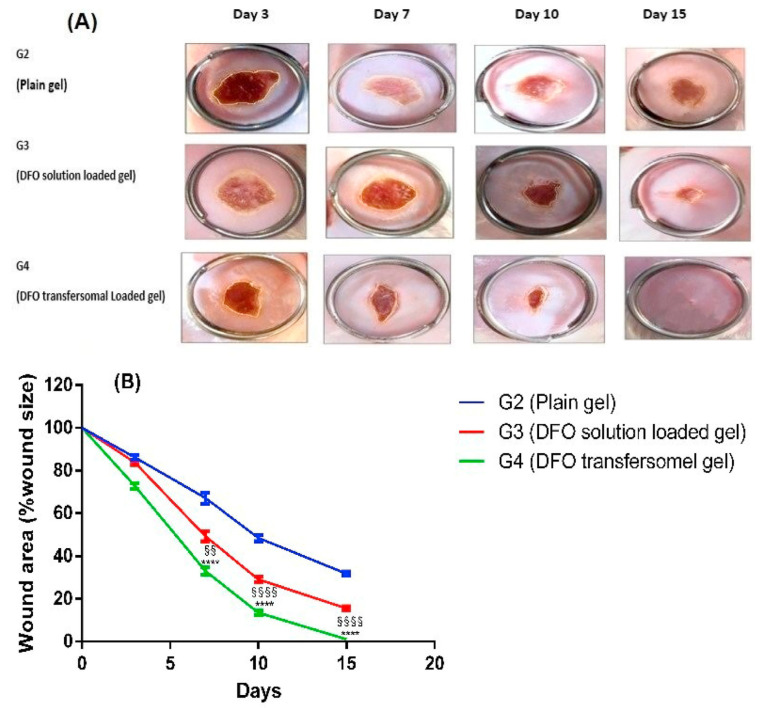
(**A**), Digital photographs of the wounds at days three, seven, and 15 after treatment. Group 2 (G2) received plain gel and served as a positive control. (G3) Received DFO (Deferoxamine) solution loaded gel once daily. (G4) Received DFO transfersomal loaded gel once daily; (**B**), Quantification of the wound area was performed using Image-J software (version 1.52n). Data are expressed as mean ± S.D. (*n* = 6), **** *p* < 0.0001 vs. G2, ^§§^ *p* < 0.01 vs. G3, ^§§§§^ *p* < 0.0001 vs. G3 [179]. Copyright 2020 Elsevier.

Due to the specifics of transfersome application, aqueous solutions of nanoparticles are not quite suitable for transdermal administration due to their inability to stay on the skin surface for a long time. This limits the effectiveness of drug delivery to the target sites. Therefore, most of the created transdermal formulations are made in the form of gels, which facilitates the application of these compositions and makes it possible to compare them with commercially available gels and creams. Carbopol 934 [184], hydroxypropyl methylcellulose [185], or even mixtures of Carbopol 971P, Poloxamer 407, and Prosopis Africana peel powder are most commonly used for this purpose [186]. In [179] sodium carboxymethyl cellulose was chosen to increase the viscosity of transfersomes solution. The optimized formulation of deferoxamine-loaded transfersomes showed excellent results in the treatment of cutaneous wound in rats’ diabetic pressure ulcers (Figure 10).

Ultra-deformability is a distinctive property of transfersomes and a key point in the choice of their application area compared to other nanoscale delivery systems. Recently, a huge number of reviews and research papers have been published confirming the efficacy of the ex vivo use of transfersomes [187,188,189,190]. However, based on the in vivo studies, these nanocarriers can undergo destructive effects in the organism, and the bioavailability of drugs can decrease.

One of the most complex forms of skin lesions is cutaneous leishmaniasis. The drug for this ailment can be taken both orally and topically. The inclusion of miltefosine in a transfersomal gel to increase its bioavailability was an effective way. The improved system was not only safe in contact with animal skin (even after deformation/reformation), but also showed improved anti-leishmaniasis activity in vivo compared with pure drug solution [191].

In another work, a transfersomal gel was obtained for the treatment of leishmaniasis with coadministration of two drugs, rifampicin and vancomycin. Important parameter of obtaining transfersomes for the leishmaniasis treatment is their size. They should not be smaller than 100 nm because they avoid uptake by macrophages. The results of in vivo experiments have shown that topical use of transfersomal gel leads to a reduction in infection compared with individual drug gels and aqueous transfersomal solutions [192].

Along with an assessment of transfersome penetration through the skin, it is important to evaluate their safety in contact with the skin. In [165] it was shown that a nanoemulsion form of transfersomes with phosphatidylcholine, ceramide III and Tween 20 effectively delivered retinyl palmitate to deep skin layers of pig ears. The compatibility of the transfersomes obtained in the emulsions with the skin was then demonstrated on a group of volunteers. It was found that the formulated systems do not affect the integrity of the skin and are safe.

As mentioned above, the main task of developing lipid nanocarriers is increasing the drug bioavailability. Many drugs have poor oral bioavailability and their inclusion in transfersomes is an alternative to increase their efficacy. An example of such drug is felodipine used to treat hypertonia and angina pectoris. The commercial drug Plendil^®^ and a dry transdermal gel based on sodium deoxylate and phosphatidylcholine at ratio of 5:1 were tested in [193]. In vivo experiments were performed on three groups of rabbits and pharmacokinetic analysis of their blood plasma was evaluated. It was found that administration of felodipine in transfersomes leads to increased C_max_ and AUC_0-48_ in plasma compared with oral administration (C_max_ = 102.6 ± 11.3 and 61.9 ± 21.9 ng/mL respectively, AUC_0-48_ = 2502.9 ± 314.3 and 1498.1 ± 205.3 ng/h/mL, respectively). It should be noted that the time required to reach the maximum concentration of felodipine in the blood does not differ much for transdermal (12 h) and oral delivery (11.3 h). Similar results were obtained when a migraine drug with poor oral bioavailability, sumatriptan succinate, was developed as a transdermal gel [194]. The bioavailability of the drug increased 4.09-fold compared with oral administration. Results obtained suggest the necessity of including the drugs in transfersomes. This helps to increase the concentration of the substrate directly in the blood, avoiding presystemic elimination in the gastrointestinal tract.

Berberis aristata extract is widely used to treat skin diseases, and its encapsulation in transfersomes has shown promising results. An experiment was conducted in several directions on albino rats, investigating its anti-inflammatory activity and effect in the treatment of psoriasis. For the in vivo experiments, paw edema and psoriasis were artificially induced in rats by administration of carrageenan and imiquimod, respectively. The edema inhibition rate for the transfersomal gel based on phosphatidylcholine and Span 80 at ratio of 80:20 was 55.76%, while for the conventional gel it was only 33.5%. According to the histopathological results, the transfersomal gel with Berberis aristata extract indeed resulted in a reduction in epidermal thickness, i.e., reduction of psoriasis, compared with the control group [195]. A similar concept was developed by another group, and these researchers used the transdermal delivery of emodin, an ingredient of Chinese medicinal herbs, that promotes weight loss [196].

The research is not limited to transdermal administration of transfersomes. Promising results were obtained with intranasal administration of a transfersomal mucoadhesive gel containing resveratrol (RES), which is an antioxidant used for the management of several central nervous system diseases [197]. The transfersome effectiveness was assessed by pharmacokinetic analysis of rat blood plasma. The results showed that the maximum concentration of RES in plasma was 2.15-fold higher than that of free RES administered orally. A stable concentration of the drug encapsulated in transfersomes in plasma was observed at 24 h, while in oral administration the drug was eliminated by 8 h of experiment. This group of scientists carried out a comparative evaluation of the RES delivery effectiveness across the blood-brain barrier in Alzheimer’s disease by transfersomes and nanoemulsions modified with gold nanoparticles [198]. The efficiency of RES encapsulation in the case of gold nanoparticle-modified nanoemulsions was higher compared with transfersomes, 95.72 ± 5.34% and 69.53 ± 3.82%, respectively. But better permeability of the drug through nasal mucosa was shown for the transfersomal system. To evaluate the effectiveness of RES delivery to the brain with subsequent memory recovery in rats, an experiment with the Morris water maze was used. It was revealed that the group of rats treated with transfersomes showed the highest time spent in the target square with a slight difference from the normal group, indicating memory recovery in the rats. Computer tomography was used as an additional experiment to visualize particles in the brain. The images of the rat brain treated with transfersomes showed a greater intensity of luminescence, which corresponds to the accumulation of gold nanoparticles included in transfersomes.

Drugs with poor oral bioavailability can be administered transbuccally by mucoadhesive gels. A transferomal gel with three different nonionic surfactants (Tween 80/Span 60/Span 80) was created and loaded with the poorly soluble antihistamine drug loratadine. Effectiveness of transfersomal loratadine was compared with commercial oral Claritin^®^ tablets. Although ex vivo loratadine passed through the skin better in the transfersomal formulation, in human experiments the difference in C_max_ and AUC was statistically insignificant, which suggests the need of optimization the transfersome formulation [199]. Another group of scientists created oral dispersible tablets based on the polysaccharide polymer pullulan with transfersomal rosuvastatin. Dispersibility tests were also performed on a group of volunteers and a pharmacokinetic study on Wistar rats. Pullulan-based transfersomal tablets were dispersed in the mouth in 1.762 ± 0.907 min, which met the pharmacopoeia standards for tablets. According to the pharmacokinetic results, the C_max_ and AUC_0–t_ values of rosuvastatin for the dispersible tablets were higher compared with the commercial tablets (C_max_ = 1102.67 ± 44.52 and 878 ± 18.25 ng/mL, AUC_0–t_ = 37,669 ± 1776 and 28,197 ± 1726 ng/h/mL, respectively). Therefore, the obtained systems may indeed improve the bioavailability and therapeutic activity of rosuvastatin in further clinical trials [200].

Transethosomes consist of phospholipid, ethanol, water and edge activator (Tween 20, Tween 80, Span 20, sodium taurocholate, sodium deoxycholate) or permeation enhancer (for instance, oleic acid). The issue of transethosomes was raised in several reviews [153,172,201,202]. In our review, we will focus on in vivo experiments with transethosomes. In general, transethosomes have a negative charge due to the edge activator. A promising approach is the use of cationic surfactants (for example, CTAB) to impart a positive charge to the surface of transethosomes [203,204] in order to increase the affinity of the nanocontainer to the negatively charged cell membrane. The presence of ethanol can lead to both a decrease [204] and an increase [167] in the size of the nanocarriers. In this issue, there is no clear tendency. The range of substrates loaded into transethosomes is quite wide and is determined by the need to use the transdermal route of administration.

It has been shown that transethosomes with oleic acid have better characteristics compared with Tween 80 and sodium taurocholate [167]. These transethosomes were loaded with voriconazole, and their TEM photographs showed a deviation from the spherical shape, which implies a higher fluidity due to the disrupted lipid bilayer. As determined by HPLC, the amount of voriconazole in the epidermis/dermis was significantly higher compared with conventional liposomes and transfersomes. In [204], transethosomes had a smaller size but greater encapsulating efficiency of paeonol compared with transfersomes. An enhanced transdermal flux of 95.7 ± 8.8 μg/cm^2^/h and a higher deposition quantity in porcine ear skin were shown for paeonol-loaded transethosomes compared with the transfersomes. The AUC of the paeonol-loaded transethosomes was approximately 1.57-fold higher than those of the transfersomes.

A promising approach is the formation of a hydrogel composition based on transethosomes. In [205], gels containing a vesicle dispersion of piroxicam, Carbopol 934, triethanolamine, and distilled water with variable amounts of lipids and ethanol were obtained. The size of transethosomes was large (550–800 nm), with a polydispersity index (PdI) found in the range between 0.261 and 0.385. The optimized transethosome composition, showing good skin retention and drug penetration, had a vesicle size of 650 nm. Ketoconazole-loaded transethosomes were also large in size [206]. The encapsulating efficiency of optimized transethosomes was twice as high as for conventional liposomes (68.35% and 35.0%, respectively). Agomelatine-loaded transethosomes [207] showed low vesicle size (156.8 nm), high entrapment efficiency (78.57%) and enhanced transdermal flux (18.75 μg/cm^2^/h). An optimized transethosome composition was loaded into Carbopol 934-based gel. Enhanced permeation of rhodamine B containing gel to the deeper layer of skin was confirmed by confocal laser scanning microscopy. Histopathology of mice brain revealed that transethosomes with agomelatine may help to recover disrupted neuroplasticity of hippocampus. Transethosomes containing progesterone and additionally modified with CTAB were incorporated into a gel using Carbopol 974, hydroxyl propyl methylcellulose and sodium alginate [203].

Dermal delivery of ferrous chlorophyllin (Fe-CHL), a photosensitizer, via transethosomes (EE = 50–40%, d = 300–700 nm, ZP = −40/−50 mV) for treatment of melanoma by PDT was discussed [208]. The selected composition was incorporated in a gel containing 2% (*w*/*w*) Carbopol 934, and small tumors were successfully cured within one month after a single PDT. This indicates the effectiveness of the combination of Fe-CHL with a light dose of 720 *J*/cm^2^ for a selective photodynamic response at the site of the tumor. Large tumors completely regressed within two months in eight mice after a single PDT treatment. 

In [209], (−)-epigallocatechin-3-gallate, a promising nutraceutical for skin carcinogenesis treatment, was loaded in transethosomes with the size of 238 nm, with a slightly lower EE% compared with ethosomes (57% and 74.68%, respectively). The antioxidant activity and photostability of the drug is saved by transethosomes. The resulting formulation exhibited an inhibitory effect on epidermoid carcinoma cell line (A431) and reduced the tumor sizes in mice.

The use of transethosomes to enhance the transdermal delivery of olmesartan medoxomil, an antihypertensive drug, was shown in [210]. The best results were obtained using sodium deoxycholate as an edge activator (EE = 58.50% ± 1.30%, d = 222.60 ± 2.50 nm, PdI = 0.11 ± 0.06, ZP = −20.80 ± 0.30 mV). A dermatokinetic study showed significantly higher C_max_ and AUC_0–10_ for transethosomes compared with drug suspension. Thus, tranfersomes and transethosomes can be successfully used for transdermal drug delivery, as shown by the in vivo experiment.

#### 3.1.3. Chitosomes

The decoration of liposomes with various polymers, in particular with biopolymers, is especially relevant of late. Polymers can improve the in vitro/in vivo stability of liposomes and reduce the drug release rate. This is due to the fact that the polymers provide a hydrophilic steric layer around the liposome [211]. Among an enormous number of polymers currently available, chitosan (CS) is widely used to modify the liposome surface [212,213,214]. For a long time, CS was the focus of researchers due to its unique properties, such as lack of toxicity, low immunogenicity, antimicrobial activity, biocompatibility, and biodegradability. CS and its derivatives can significantly enhance the transdermal drug delivery rate [215,216]. The positively charged amino groups of CS most likely interact with the negatively charged components (anionic phosphates, carboxylates, etc.) of skin, thereby improving the diffusion of the drug into its deeper layers [215]. The authors of [217] investigated the mechanisms of interaction of CS with the surface of the mice epithelium. CS and related derivatives may have significant impact on the secondary structure of keratin, increase the water content in the SC, reduce the potential of the human immortalized keratinocyte cell membrane and increase its fluidity. Such liposomal complexes with CS are called chitosomes [218]. CS can interact with the negatively charged liposome by an electrostatic mechanism due to the CS positive charge. Another mechanism, such as hydrogen bonding between the polysaccharide and phospholipid head groups, may also be involved in complexation. Also, CS reduces membrane fluidity, limits liposome aggregation, and increases the steric stability of the resulting nanostructures [219]. The above-described properties of CS make chitosomes more preferable over traditional liposomes. The possibilities of using chitosomes for transdermal delivery have been extensively investigated and summarized in a review [220].

In most cases [221,222] the coating of the liposome with CS is carried out in two stages. At the first stage, liposomes containing a substrate are formed, and then the liposome solution is mixed with the CS of the required concentration with constant stirring. It should be noted that it is necessary to carefully select the concentration and ratio of the components to obtain stable particles. However, there are examples of chitosomes preparation in one stage. This can be achieved by injection of the organic phase containing the lipid into an aqueous solution of CS with constant stirring [223].

**Figure 11:**
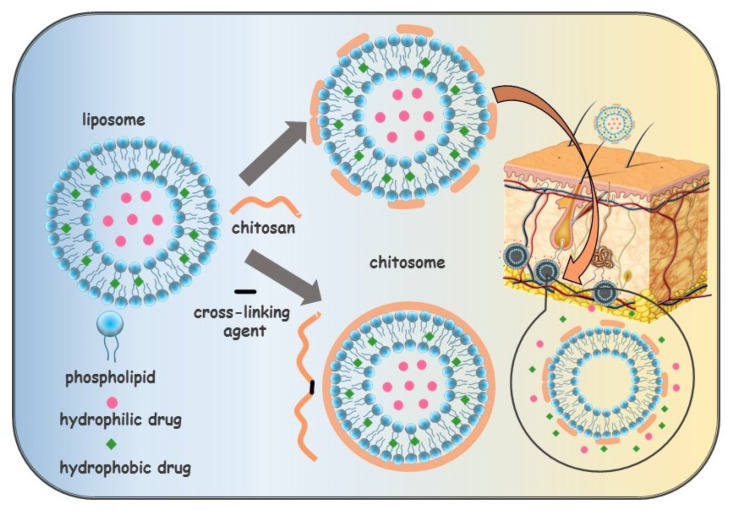
Scheme of obtaining chitosan-coated liposomes.

Schematic representations of obtaining chitosomes are presented in Figure 11. As can be seen from this figure, the interaction of chitosan with the surface of liposomes can be because of electrostatic interactions between the oppositely charged head group of phospholipids and the CS amino group. Also, CS can completely cover the liposomes due to the addition of a cross-linking agent (alginate, citrate, sodium tripolyphosphate) [224]. A stable 3D network (ionic gelation) is formed around liposomes based on electrostatic interaction between protonated amino groups of CS and anionic groups of the cross-linking agent [225]. It is worth considering that the addition of CS leads to an increase in the size of liposomes and a recharge of their surface [226,227,228].

The interaction efficiency between components of chitosomes depends on various factors: lipid structure, medium parameters (pH, ionic strength), molecular weight, concentration, deacetylation degree, charge and CS modification [229,230,231,232]. CS can be modified due to the presence of amino and hydroxyl groups. This modification can significantly improve the properties of CS and expand the practical application of chitosomes. For example, CS is poorly soluble in water, which makes it difficult to use it for decorating liposomes [233,234]. N-stearyl chitosan with a molecular weight of 41 kDa was used as a coating material for liposomes containing the antifungal and antiviral drug itraconazole (EE% was over 90%) [235]. A significantly slower release of itraconazole encapsulated in liposomes coated with N-stearoyl chitosan was shown when compared with the free drug. The authors of [234] prepared multilayered liposomes containing quercetin, and these vesicles were coated with chitooligosaccharide (0.8–3 kDa) and N-succinyl-chitosan. The release behavior of quercetin from multilamellar liposomes was evaluated at a neutral pH of 7.4 and at normal skin pH of 5.5. The results of an in vitro experiment showed that the amount of released quercetin tends to a decrease with an increase in the number of polymer layers, and was higher at a pH of 5.5. Interestingly, four-layered liposomes were the most stable with Triton X-100 treatment. According to the in vitro skin permeability study results (using Franz diffusion cells), four-layered liposomes have a higher skin penetration degree than uncoated liposomes. The authors suggested that polyelectrolytes retain water, which hydrates the SC, and enhances the quercetin release at pH 5.5.

The polymer chain length has practically no effect on the physicochemical properties of chitosomes [232]. However, it can influence the surface charge of liposomal formulations, determining stability and behavior in the human organism. In [231], using isothermal titration calorimetry, the effect of pH on the interaction of low molecular weight CS and 1,2-dioleoyl-sn-glycero-3-phosphocholine (DOPC) liposome was studied. The values of pH ≥ 6.0 are the best for interaction between components. Under these conditions, CS deprotonation increases, which leads to its stronger adsorption on the liposome surface, as evidenced by high exothermic heat release. It is assumed that on the liposome surface the conformations of the polymer chains differ from the conformations at lower pH.

CS exhibits anti-tumor activity by inhibiting cellular metabolism, thereby causing suppression of cell growth. Due to this property, chitosomes can be potential carriers for a large number of chemotherapeutic agents. The authors of [226] demonstrated the possibility of using chitosomes as containers for stabilizing and enhancement of the indocyanine green (ICG) penetration through the skin. ICG is a promising candidate for the topical photodynamic therapy of melanoma. However, its significant drawback is the inability to penetrate into deep skin layers due to its hydrophilic nature. The authors showed that positively charged chitosomes promote cellular uptake and photocytotoxicity in B16-F10 melanoma cells. Interestingly, large chitosomes (>1000 nm) containing ICG penetrate the skin significantly better than unmodified liposomes (257 nm). Thus, chitosan coated liposomes overcome the size limitation when penetrating through the skin due to various mechanisms of increasing permeability.

The authors of [236] presented chitosomes with the following composition: 1,2-dipalmitoyl-sn-glycero-3-phosphocholine (DPPC), 1,2-distearoyl-sn-glycero-3-phosphoethanolamine (DSPE), cholesterol and CS with different ratios containing the antitumor drug metformin hydrochloride. These systems had increased cellular internalization, which led to an increase in the cytotoxicity on mesothelioma cells and to a decrease in the chemotherapeutic dose of the drug. The results obtained confirm the effectiveness of metformin-containing chitosomes, which can act as an alternative treatment for malignant pleural mesothelioma. The authors recommended these delivery systems for preclinical studies.

Microorganisms (bacterial and fungal species) pose a serious threat to human health. The most frequent superficial skin lesions lead to serious diseases (vaginitis, dermatitis, mycosis, etc.). Conventional antibacterial and antifungal formulations have a number of limitations, such as short residence times, instability, poor water solubility, and difficulties in penetration through biological membranes. This prevents their use in transdermal drug delivery. However, liposomal systems decorated with CS can by themselves have an antifungal effect and also act as a nanocontainers for antimicrobial drugs [235,237,238]. In [237], the authors encapsulated the antimicrobial drug metronidazole in chitosomes. To prove that the mucoadhesiveness of chitosan alone is insufficient for successful delivery, Carbopol-containing liposomes were formed as a control. It was shown that chitosomes provide a prolonged release of metronidazole and have a strong ability to inhibit the growth of *Candida albicans*. The antifungal efficacy of chitosomes combined with the antibacterial activity of encapsulated metronidazole may provide increased efficacy in the treatment of mixed/complex vaginal infections.

Physicians are very often faced with the problem of biofilm formation and increased resistance to antimicrobial drugs in the treatment of chronic wounds, causing delayed healing. To solve these problems, an antimicrobial system based on liposomes, which is incorporated into a CS hydrogel, was developed [238]. The model of the membrane-active antimicrobial drug chlorhexidine was chosen as the substance. Liposomal chlorhexidine (~50 μg/mg of lipids) in chitosan hydrogel significantly suppressed the production of nitric oxide in macrophages induced by lipopolysaccharides and almost completely reduced biofilm formation. Moreover, it inhibited the growth of *Staphylococcus aureus* and *Pseudomonas aeruginosa* cells in biofilms by 64.2% and 98.1%, respectively, while empty liposomes were inactive. It is important to note that the inclusion of liposomes in CS hydrogel improved the antibacterial effect of chlorhexidine. This is demonstrated by a decrease in the minimum lethal concentration for all tested strains.

It is known that some commercial products have a number of disadvantages. For example, conventional sunscreens (lotions, creams) cannot penetrate into the deeper layers of the skin and remain on them for a very short time after topical application. Sunscreens in liposomal carriers can prevent skin irritation and enhance photoprotection by increasing the photostability of the active ingredient [239]. In [240], chitosomes based on the commercial lipid Phosal^®^ 53 MCT, nonionic surfactant Tween 80, and low-molecular weight CS, containing the UV sunscreen octyl methox-ycinnamate (EE% of over 94%), were obtained. It is speculated that CS can form a homogeneous film due to its cationic nature, keeping the sunscreen on the skin. Chitosomes showed higher SPF values (9.7 to 10.3) and delayed in vitro release of octyl methoxycinnamate than unmodified liposomes (SPF 7.3). It was found that an increase in the concentration of CS to 0.5% leads to an increase in the cytotoxicity of the entire nanoformulation toward skin fibroblast cells and keratinocytes (HaCaT), and at a concentration of ≤0.3% it remains acceptable. The authors of [241] proposed a liposomal system based on soybean phosphatidylcholine and sodium deoxycholate and coated with CS as a carrier for transdermal administration of terbutaline sulfate (TBN), used to improve lung function. Commercial TBN has low oral or inhalation bioavailability. Optimized TBN-containing chitosomes were spherical vesicles (245.13 ± 10.23 nm) with EE% equal to 65.25 ± 5.51% and had good skin permeability (Male Wistar rats) ex vivo (340.11 ± 22.34 μg/cm^2^). Histopathological examination showed that the resulting composition was well tolerated without signs of inflammation. Pharmacokinetic studies showed that the liposomal formulation consistently increased TBN bioavailability (2.33-fold) and elimination half-life (up to 6.21 ± 0.24 h) compared to TBN oral solution. The authors of [227] developed an effective transdermal delivery system based on soybean lecithin and CS for the local anesthetic lidocaine hydrochloride. The ability of chitosomes to penetrate through the skin under in vitro and in vivo conditions was assessed. The EE% of drug decreased with an increase in the CS content. In another work, CS-coated liposomes were used as an effective transdermal RES delivery system to slow aging and provide antioxidant protection of the skin [228]. The amount of RES permeating through the animal skin was 40.42% and 30.84% for coated and uncoated liposomes, respectively. In [239] liposomes of phosphatidylcholine from soybean coated with low-molecular-weight, CS (50 kDa) were used as a potential delivery system for alpha-lipoic acid and coenzyme Q10. Chitosomes containing these antioxidants exhibited low cytotoxicity and good antibacterial (against *Escherichia coli* and *Staphylococcus aureus*) and antioxidant activity.

CS can be modified with cell penetrating peptides. In [242], a CS derivative, N-arginine-chitosan was used as a penetration enhancer for transdermal delivery of the antiviral drug adefovir. The best results were obtained using CS with a molecular weight of 10 kDa at a concentration of 2% (*w*/*v*) and with a degree of substitution of CS with arginine equal to 6%. Other authors showed that chitosomes modified with arginine (chitosan-arginine-DOTAP/DOPE system) are able to transfect DNA into HEK293 T cells as efficiently as lipofectamine (a commercial drug, transfection efficiency ~90%) [243]. Most likely, the small size (116 nm) and high zeta potential (+52 mV) of chitosomes promote efficient interaction with DNA without causing its aggregation and an increase in size. It was shown that chitosomes form multilayer vesicles with DNA, and the DNA molecule is located between the bilayers, because of which the DNA is well protected from lysosomal enzymes. These results indicate that chitosomes are promising carriers for in vivo gene delivery. N-Octyl-N-arginine-chitosan (OACS) was used to coat liposomes as an absorption enhancer for cyclosporin A [244]. The OACS hydrophobic octyl group is incorporated into the phospholipid bilayer of the liposome through a hydrophobic bond. OACS can slow drug release and protect its degradation in the stomach. In situ single pass perfusion proved that absorption of OACS-coated liposomes loaded with cyclosporine A in the small intestine was increased 3-fold and 22-fold compared with the commercial microemulsion Tianke and the pure drug suspension, respectively.

#### 3.1.4. Invasomes

**Figure 12:**
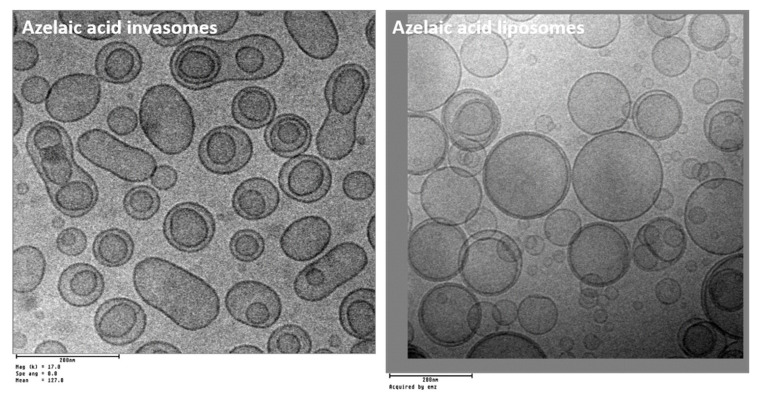
CryoTEM images of invasomes and liposomes loaded with azelaic acid [253]. Copyright 2015 Elsevier.

Invasomes are flexible or deformable vesicles containing three components: phospholipids (phosphatidylcholine, phosphatidylserine, phosphatidylinositol, etc.), ethanol and terpene or a mixture of terpenes (citral, cineol, limonene, eugenol, etc.) [245,246,247]. A bulk of the articles on these new lipid formulations was published over the past five years. In contrast to chitosomes, invasomes as carriers are used only for transdermal drug delivery. However, alcohols and terpenes were known much earlier as penetration enhancers [248,249,250,251]. The presence of these particular components determines the deformability of invasomes [246,252]. For instance, cryoTEM images of invasomes and conventional liposomes loaded with azelaic acid can be seen in Figure 12 [253]; invasomes are smaller in size and have a deformed structure, as also affirmed in [254].

Terpenes have been described as transdermal penetration enhancers for nicardipine [248,249], itraconazole [255], benznidazole [256], hydrocortisone, carbamazepine, and tamoxifen [248]. The concentration of ethanol and terpene in the invasomes is also important, because in most cases action of invasomes is determined by this characteristic [257]. Terpene concentration generally ranges from 1 to 5% *v/v*, and the FDA classifies terpenes as safe for skin at these concentrations [245,250]. In the case of ethanol, its concentration does not exceed 10% *v/v* [247]. The authors of [258] used different ethanol concentrations with a commercially available lipid mixture NAT 8539 to improve the topical delivery of cyclosporine A. Vesicles containing 10% ethanol have shown a higher penetration degree of cyclosporine A into human abdominal skin compared with liposomes without ethanol. A high concentration (~40%) of ethanol leads to pore formation and significantly increases the transport of drugs through the pores. However, as ethanol concentration increases, the size and effect of invasomes decrease. This can be linked to the fact that high ethanol concentration reduces vesicular membrane thickness and ultimately vesicle size, which leads to dissolution of the invasome [245]. The increase in permeability caused by ethanol is associated with two effects: (a) push effect due to evaporation of ethanol; and (b) a pool effect due to the reduction of barrier properties of SC by ethanol [258]. The reduction of barrier properties is related to the interaction of ethanol with SC lipids, which leads to fluidization and destroys their highly ordered structure [259]. Ethanol is also used in such formulations to improve solubility of water insoluble drugs or as a co-solvent [260]. It should be noted that the improved efficiency of invasomes is a result of the synergistic effect of all components (phospholipids, ethanol, terpenes), not individual ones.

**Figure 13:**
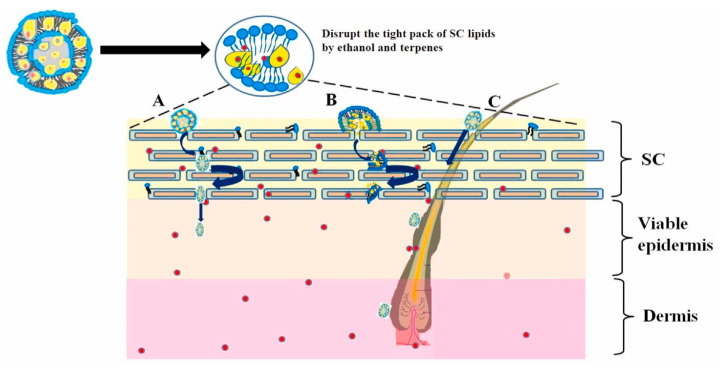
Possible penetration mechanisms of invasomes through the stratum corneum: (**A**) enhanced penetration, (**B**) intact penetration and (**C**) trans-appendageal penetration [245]. Copyright 2021 Elsevier.

There are two possible penetration mechanisms of invasomes [245]: (1) the invasomes themselves can be importer systems for drugs; and (2) terpenes can increase drug penetration by disrupting the lipid packing of SC [245,259]. It is assumed that part of the invasomes breaks down during penetration through the upper skin layers, thus releasing constituent components (individual phospholipid molecules as well as terpenes) that liquify the intercellular lipids of SC. Small invasomes can penetrate into the deeper layer of SC intact through the hydrophilic channels present in the intercellular space or follicular transport pathway (Figure 13) [245,254].

The formation procedure of invasomes and liposomes is similar: the first stage is the preparation of dry lipid film containing drug and terpenes; the second stage is the hydration of the lipid film in a mixture of ethanol and buffer (usually phosphate buffer). Particle size is a key parameter in transdermal drug delivery. Therefore, sonication and extrusion are often used for obtaining monodisperse vesicular systems with small diameters. The size of invasomes increases with the concentration and molecular weight of terpenes. In [261], the size of isradipine-loaded invasomes containing 0.1% b-citronellene was about 194 nm, while being equal to 230 nm at a concentration of 0.5%. The size of nimesulide-loaded invasomes containing citral, limonene, and cineol was 194 nm (PdI 0.26), 216 nm (PdI 0.17), and 244 nm (PdI 0.1), respectively [262]. Dragicevic-Curic et al. demonstrated the morphology of invasomal vesicles containing temoporfin by the cryoTEM. The vesicles with 1% terpene mixture (cineole, citral and D-limonene) or only one terpene (citral) were mostly unilamellar and spherical with a diameter of about 100 nm [254]. It should be noted that the invasome size also depends on the drug loaded. For example, the size of tolterodine tartrate-loaded invasomes containing 1% limonene, phenchone, and anethol was about 1.3 μm (PdI < 0.2) independent of the terpene structure [263].

Temoporfin is a powerful synthetic photosensitizer, which is effective in the photodynamic therapy of skin tumors. Unfortunately, it is a highly hydrophobic drug which is poorly absorbed through the skin. Dragicevic-Curic et al. investigated the photodynamic efficacy of temoporfin-loaded invasomes after their application onto mice skin with implanted human colorectal tumor HT29. Survival patterns of groups of mice treated with temoporfin-loaded invasomes containing 1% terpene mixture before photodynamic therapy showed significantly less (*p* < 0.05) tumor growth compared with control groups (mice without any treatment and mice only after photodynamic therapy) [254]. Qadri et al. [261] fabricated isradipine-loaded invasomes with different composition for the potential treatment of hypertension. The best results were shown for the optimized invasome formulation composed of the following components phospholipid:ethanol:citrenol with the molar ratio of 2%:10%:0.1%. This formulation presents maximum entrapment efficiency (89%) and transdermal flux (22.80 ± 2.10 mg/cm^2^/h) through rat skin in vitro. In a pharmacodynamic study, the isradipine-loaded invasomes were converted into gel for ease of application and longer skin contact. The blood pressure of rats with hypertension induced by administration of deoxycorticosterone acetate was monitored for 24 h. Application of the isradipine-loaded invasomal transgel resulted in a 17.43% steady lowering of blood pressure after 4 h. It was observed that usage of the invasomal gel has maintained a normal value of blood pressure for up to 24 h.

The authors of [253] compared three vesicular systems: cationic LeciPlex (based on CTAB and DDAB), invasomes, and liposomes for their ability to deliver drugs deep into the skin. Drug-loaded LeciPlex and conventional liposomes were about 300 nm in size with the PdI values between 0.2 and 0.4, while invasomes were around 140 nm with the PdI of 0.09. Ex vivo human skin penetration studies showed that LeciPlex formulations demonstrated higher penetration degrees for idebenone, whereas invasomes showed better penetration of azelaic acid. LeciPlex formulations containing idebenone had a superior activity against B16F10 melanoma cells, followed by invasomes and liposomes. In the antiacne efficacy study in rats, invasomal azelaic acid demonstrated high efficacy, followed by liposomes and LeciPlex. 

Invasomes containing tolterodine tartrate (TT) were prepared using soy lecithin (1–7%), ethanol (10%) and three different terpenes (limonene, phenchon and anethol, 1%) [263]. Vesicles were stable for two months at +4 °C and more than 90% of TT was retained in the invasomes. The ex vivo skin penetration data showed that the invasomal drug dispersion penetrated significantly better through the rat skin compared with vesicles without terpenes, ethanol TT solution and TT solution. Better penetration through the skin was observed for the invasomal formulation containing limonene, but worse with phenchone. This is due to the high lipophilicity and low boiling point of limonene, which is confirmed by other authors [248]. The low boiling points of terpenes indicate weak cohesiveness or self-association of the molecules and, thus, they can more easily interact with the SC lipids and change the barrier properties. The results of iontophoresis showed higher permeability of encapsulated drugs compared with the free one and proved the additive effect of invasomes and iontophoresis.

Avanafil (AVA)-loaded invasomes, based on Phospholipon^®^ 90G, ethanol and terpenes (limonene, citronellol) were developed and optimized by the authors of [257]. The percentage of phospholipids has a significant effect on both vesicle size and drug loading. Terpene type was highly affected the invasome size, while terpene concentration affected AVA loading. A hydroxypropyl methylcellulose-based transdermal film containing optimized AVA-loaded invasomes (10.47% phospholipid, 2.00% ethanol, and 1.50% D-limonene) demonstrated improved ex vivo permeation compared with the raw AVA film. Furthermore, in vivo pharmacokinetic evaluation in rats showed a higher bioavailability of optimized AVA film.

The brief evaluation of different types of nanocontainers in terms of their advantages and limitations allows us to presume that, unlike with micellar and microemulsion systems that are dynamic structures, liposomes are stable over time nanoparticles with relatively unchanged molecular composition. Liposomes are biologically inert, completely biocompatible and practically do not cause toxic or antigenic reactions. It is also important, that medicinal substances, encapsulated in liposomes, are less toxic, protected from the destructive action of external factors, and have better pharmacological parameters [121,122,264]. Characterization and assessment of the stability of the resulting product is undoubtedly of practical importance at the stage of liposome development. For this purpose, three main indicators are generally used—vesicle size, polydispersity index and ζ-potential [265]. It should be noted that the stability of liposomes in vitro might differ from the stability of liposomes in vivo. In vivo, liposomes can be rapidly cleared from the blood and taken up by cells of the reticuloendothelial system. Most liposomes are assimilated by the phagocytes of cells through endocytosis and degraded in lysosomes [266]. This topic has remained as an urgent challenge over an extended period. Several generations of liposomes were developed to achieve the in vivo stability of liposomal drugs, including stealth liposomes decorated with a polyoxyethylene mantle and immunoliposomes modified with antibodies, etc. [267,268,269]. Importantly, other vesicular formulations discussed in this review, namely niosomes, transfersomes, and invasomes, to some extent can be considered as further development of liposomal formulations adjusted to specific tasks. Their stability and tools for its improvement are very similar. This is obviously exemplified by consideration of chitosomes as one of the effective ways to combine the advantages of liposomal carriers with biological resistance of chitosan. Importantly, the universal tool proposed for the improvement of in vivo stability of all the vehicles discussed is noncovalent modification with cationic surfactants, which has been documented to prevent particle aggregation and provide a targeting effect toward cell membranes.

## 4. Conclusions

To summarize, drug delivery systems based on amphiphilic building blocks were reviewed. Due to the dual character of amphiphilic molecules, they are able to self-assemble in aqueous solution with the formation of aggregates, composed of two compartments with different micropolarity. They find a wide application in a variety of technologies, which is based on their ability to bind important compounds in both hydrophilic and hydrophobic domains, thereby modifying characteristics of guest molecules. From the viewpoint of the design of drug delivery systems, amphiphilic nanocarriers have numerous priorities due to the biomimetic character responsible for their affinity toward biological species, primarily toward the biomembranes. To date, nanocarriers based on natural amphiphiles, lipids, have received much attention, with many liposomal drugs approved for commercial use. Meanwhile, with the development of liposomal medications, further specific problems have appeared, which took additional efforts from researchers aimed at the improving the liposomal vehicles and adapting them to specific diseases and specific administration routes. One of the effective ways to tune the liposome properties and functionality is their modification with synthetic surfactants. Due to the facile structural behavior of surfactants, they can provide morphological lability and a charged character to lipid nanocarriers, thereby increasing their functionality in terms of controlling the surface properties (charge, curvature), improving the electrostatic affinity to cell membranes and ionic biospecies, overcoming the biological barriers, etc. Currently, the research activity related to the design of novel surfactants has markedly increased, with much attention paid to biogenic, biodegradable, low toxic, and biocompatible amphiphiles. Therefore, a special section of the review was devoted to synthetic surfactants, including those bearing cleavable carbamate moiety and natural amino acid fragments. From the viewpoint of biomedical application, a further avenue for research would be to focus on the design of versatile amphiphilic platforms with stimuli-responsive functions and targeted moieties responsible for the construction of surface-active agents with controlled morphological behavior and addressed biological function. In this context, surfactants with the pH-dependent group, macrocyclic derivatives, metallosurfactants, and amphiphilic peptides are of special interest [5,24].

Another line of discussion focused on the design of amphiphilic nanocarriers capable of crossing biological barriers, with transdermal delivery particularly emphasized. To optimize the liposomal properties for transdermal administration, different modifying agents were used, including nonionic surfactants (transfersomes, niosomes), alcohols (ethosomes), polymers (chitosomes) and terpene derivatives (invasomes), etc. The mechanism of modification of liposomal characteristics involves the integration of additives in the lipid bilayer, thereby modifying the periphery properties, especially fluidity and deformability. This allows vesicular particles to adjust their shape upon penetration through tight channels, with drug molecules becoming entrapped. Noncovalently modified nanocarriers demonstrate marked advantages from the viewpoint of the ease in tailoring their key properties, including size, charge characteristics, loading capacity, controlled drug release, and toxicity by the optimization of their composition, which allows them to be recommended as versatile and effective nanocarriers capable of overcoming biological barriers. The transdermal route of drug delivery highlighted in this review has numerous advantages over oral or intravenous administration (simplicity, painlessness, the possibility of prescribing this form to patients who have difficulty chewing and swallowing). Nevertheless, there are many tasks to be solved, e.g., nanocarriers for transdermal delivery, and like any system they have their own drawbacks; so far, these include insufficiently overcoming the stratum corneum and the difficulty of achieving high drug concentrations to increase their therapeutic effect. In this case, a promising way is the combination of nanocarrier strategy and physical methods for improved drug delivery through the skin, for example, iontophoretic delivery, the use of ultrasound and microneedles [270,271]. In [272], the use of ethosomes in combination with sonoporation and electroporation improved transdermal delivery of calcein. Such combinations appeared to be more effective than by simply using a liposomal system. It is worthy of note that nanoscale transdermal delivery systems have found a place in many areas of medicine, and their usage is not limited to the treatment of local skin diseases or inflammatory processes. There are studies on the application of such systems for the treatment of diabetes and cardiovascular diseases [273].

Much effort has been focused on the optimization of the composition of nanocontainers for transdermal delivery and the search for new modifiers. It should be noted that both the fabrication of hybrid nanocarriers by noncovalent coupling and the modification of lipids through various chemical reactions are of practical interest [274]. However, the problem of their biodegradability and targeting has not yet been resolved, which opens up new vectors of research. Undoubtedly, the main task of all scientists is to commercialize such systems. Meanwhile, the path from creation to clinical practice is very long, costly, and laborious. A relatively small number of transdermal drug delivery systems in patch form are known [275]. Inclusion of liposomal systems into such matrices is currently the main and, in view of their increasing discussion in publications, rather realistic goal.

## Figures and Tables

**Table 1:** Niosomes as vehicles for drug delivery.

Composition	Loaded Drug	Route of Administration	Ref.
Span 40, 60, 80; Tween 40, 60; cholesterol	Pentoxifylline	topical drug delivery	[129]
Span 60; cholesterol; sodium carboxymethylcellulose	Insulin	transdermal drug delivery	[130]
Span 60; Tween 80; Cremophor RH40; cholesterol	Simvastatin	transdermal drug delivery	[131]
Span 20, 40, 60; Tween 20, 40; cholesterol; Carbopol 934	Timolol Maleate	ocular drug delivery	[132]
Span 40; Tween 40; cholesterol; hydrophobin-1	Doxorubicin	parenteral drug delivery	[133]
Span 60; cholesterol	Bromocriptin Mesylate	nose-to-brain drug delivery	[134]
Span 60; cholesterol; dicetyl phosphate	Withaferin–A	intraperitoneally	[135]
Span 80, 60; Tween 80, 20; cholesterol	Ondansetron HCl	trans-mucosal nasal drug delivery	[136]
Tween 65; Span 60; cholesterol; dodecyl sulfate sodium	Gemcitabine, Cisplatin	aerosolized drug delivery	[137]
Span 60; cholesterol	Doxycycline	ocular drug delivery	[138]
Span 40; cholesterol; Carbopol 934	Betaxolol	ocular drug delivery	[139]
Tween 20; cholesterol; chitosan; dicetyl phosphate	Pentamidine	nose-to-brain drug delivery	[140]
Span 60, 80; Tween 40, 80; cholesterol	Artemether	parenteral drug delivery	[141]
Span 20; cholesterol; dicetyl phosphate; ketorolacatromethamine	Natamycin	topical ocular drug delivery	[142]
Span 60; cholesterol; Cremophor RH40	Quercetin	transdermal drug delivery	[143]
